# Case Report: Maribavir for refractory cytomegalovirus viremia after renal transplantation in a child with Schimke’s immune-osseous dysplasia

**DOI:** 10.3389/fimmu.2025.1521763

**Published:** 2025-04-07

**Authors:** Jia-Shuan Huang, Hong-Kai Wang, Li-Ping Rong, Xiao-Yun Jiang, Long-Shan Liu, Liu-Yi Huang, Na Zhang, Zhi-Hui Yue

**Affiliations:** ^1^ Pediatrics Department, The First Affiliated Hospital of Sun Yat-Sen University, Guangzhou, China; ^2^ Medical College, Sun Yat-Sen University, Guangzhou, China; ^3^ Organ Transplantation Department, The First Affiliated Hospital of Sun Yat-sen University, Guangzhou, China

**Keywords:** CMV, cytomegalovirus GCV, Schimke’s immune-osseous dysplasia, children, renal transplant

## Abstract

Cytomegalovirus (CMV) is a major opportunistic pathogen in recipients of solid organ transplantation. Maribavir, a pUL97 protein kinase inhibitor, was approved for the treatment of refractory post-transplant CMV infection in the US in 2021. However, it is rarely used in pediatric patients worldwide. Here, we report the case of a Chinese boy with Schimke’s immune-osseous dysplasia (SIOD) who developed refractory CMV infection after a renal transplantation. An 11-year-old boy was hospitalized with recurrent abdominal and testicular pain 50 days after renal transplantation. Diagnoses included urinary tract infection, epididymitis, CMV viremia, stage 2 chronic kidney disease, and SIOD. After five days of treatment, his pain improved, but he developed persistent fever and shortness of breath. Blood CMV levels rose to 1.64 × 10^5^ copies/ml after one month of ganciclovir treatment. Significant bone marrow suppression was observed after combined treatment with foscarnet. Anti-rejection treatment was discontinued due to compromised immune function. On day 40, maribavir was initiated with parental consent, resulting in undetectable CMV copies within four days. The patient’s clinical status and bone marrow suppression had improved. Continuing maribavir for two weeks led to the disappearance of CMV viremia, no bone marrow suppression, and normal liver and kidney functions. This case demonstrates the successful short-term use of maribavir in the treatment of refractory CMV infection in an immune-deficient child after renal transplantation. Further studies are required to explore the efficacy and safety of maribavir in pediatric patients.

## Introduction

CMV is a major opportunistic pathogen in recipients of solid organ transplants and hematopoietic stem cell transplantation and is a major cause of morbidity and mortality during the first six months after transplantation (1). Ganciclovir (GCV) and Val ganciclovir (VGCV) are first-line treatment used in solid-organ transplantation to treat CMV infection (2, 3). Second-line options include foscarnet and cidofovir, which are used in cases of GCV intolerance or resistance and are associated with a substantial risk of toxicity, such as electrolyte abnormalities, neutropenia, and thrombocytopenia (4). Similarly, they have drug resistance issues (5–7). The treatment of refractory CMV infection remains challenging.

Maribavir, an inhibitor of CMV-specific UL97 protein kinase, was approved by the FDA in November 2021 as the first drug for treating adult and pediatric patients (12 years of age and older and weighing at least 35 kg) with post-transplant CMV infection or disease that does not respond to available antiviral treatment for CMV (8). However, there is insufficient experience regarding the current use of maribavir in pediatric patients.

Schimke’s immuno-osseous dysplasia (SIOD) is an autosomal recessively inherited, monogenic, ultra-rare multisystem disease (9), which is characterized by multisystem involvement given by skeletal, renal and immunological abnormalities (10). Its deficiency with T-cell lymphopenia leads to cellular immunodeficiency which makes it prone to viral infection (11, 12).

Clinical experiences with maribavir, especially in pediatric SIOD patients who have undergone kidney transplantation, are scarce. Here, we report a case of successful short-term use of maribavir for the treatment of CMV infection in a pediatric SOID patient after renal transplantation.

## Clinical case presentation

**Figure 1:**
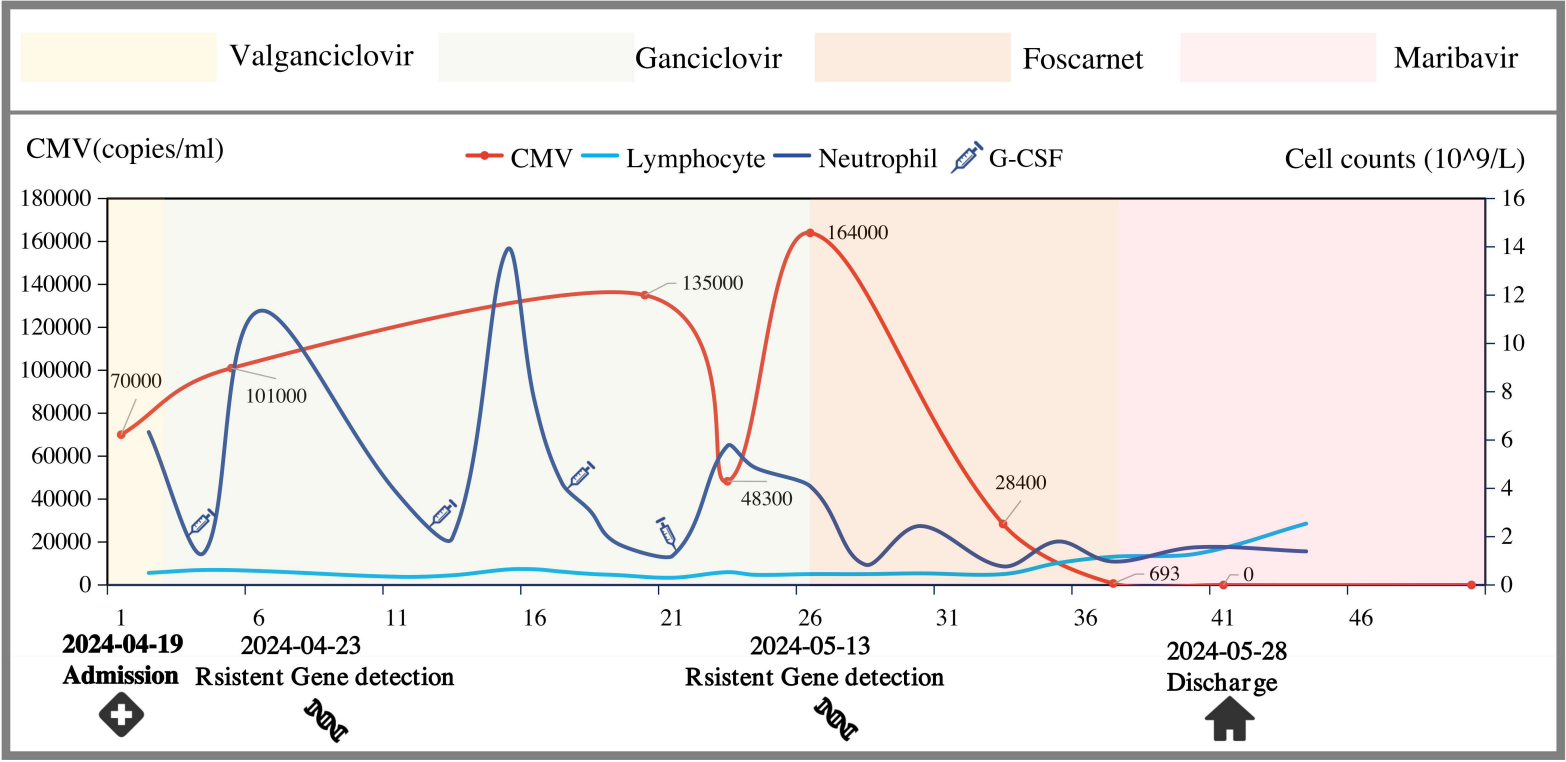
Progress of anti-CMV treatment.

An 11-year-old boy with SIOD (detailed information listed in **Table 1**) who had undergone allogeneic renal transplantation 50 days prior for end-stage renal disease was admitted to the hospital with a complaint of recurrent abdominal pain and testicular pain persisting for over one month. Tacrolimus, Mizoribine, and methylprednisolone were administered for anti-rejection therapy after renal transplantation. One month after the renal transplantation, the patient experienced recurrent abdominal and testicular pain. Urinalysis indicated the presence of *Escherichia coli*, Candida smooth, and CMV infection with CMV blood quantitative PCR (qPCR) at 7 × 10^4^ copies/ml. The patient received anti-infection therapy with piperacillin, fluconazole, and valganciclovir. However, his clinical symptoms continued to recur and was admitted to the hospital on 19 April 2024 (**Figure 1**; **Supplementary Table**).

**Table 1:** The patient’s demographic information and history.

Demographic information and history	Details
Age	11 years
Sex	Male
Ethnicity	Han Chinese
Occupation	Elementary school student
Medical History	SIOD, renal transplantation
Family History	Non-contributory
Genetic Information	*SMARCAL1* (NM_014141.3) Exon12 c.1930C>T p.(Arg644Trp) heterozygous, unknown significance; *SMARCAL1* (NM_014141.3) Exon8 c.1444delC p.(Leu482fs) heterozygous, pathogenic mutation.

SIOD, Schimke immune-osseous dysplasia.

Upon admission, the patient was diagnosed with urinary tract infection, epididymitis, CMV viremia, stage 2 chronic kidney disease, and SIOD. The patient received various antibiotics, antifungal agents, and antiviral therapy. The initial treatment with anti-CMV was daily intravenous ganciclovir and the dose was determined based on renal function. By the fifth day, the abdominal and testicular pain had significantly improved. However, the CMV blood qPCR increased to 1.01 × 10^5^ copies/ml on the 4th day after admission. At the same time, the lymphocyte count decreased to only 0.34 × 10^9^.

On the 8th day after admission, he developed fever with a gradual rise in thermal peaks, accompanied by shortness of breath. Chest CT showed interstitial pneumonia, and CMV blood qPCR persistently increasing to 1.35 × 10^5^ copies/mL on the 19th day after admission. Antiviral susceptibility testing was performed, and no resistance genes were detected. Meanwhile, a gradual decrease in lymphocytes was considered to be related to excessive anti-rejection. Mizoribine was stopped on the 14th day after admission while tacrolimus and methylprednisolone were stopped on the 21st day. Furthermore, gamma globulin was administered to boost immunity.

However, his lymphocyte count was still very low and continued to have daily fever with no significant decrease in fever peaks and intermittent shortness of breath. Alveolar lavage fluid test showed that CMV qPCR was 2.2 × 10^3^ copies/mL and CMV blood qPCR was persistently increasing to 1.64 × 10^5^ copies/mL on the 26th day after admission. Simultaneously, resistance testing was repeated. UL54 genotyping revealed the emergence of HCMV variants with mutations in S676G that confer GCV resistance (13).

According to the latest guidelines, the patient was diagnosed with refractory CMV infection, which is defined as CMV viremia that either increases (i.e., a rise of more than 1 log10 in CMV DNA levels within the same blood compartment compared to the peak viral load, as measured in the same laboratory and/or with the same commercial assay) or persists (a change of 1 log10 or less in CMV DNA levels) after at least 2 weeks of appropriate antiviral therapy (14).

Therefore, we combined the anti-CMV therapy with foscarnet. Two days later, the fever subsided and the patient’s clinical condition gradually improved. On the 36th day after admission, CMV qPCR decreased to 693 copies/ml. However, he developed significant myelosuppression with an absolute neutrophil count (ANC) plummeting to 0.77 × 10^9^/L, and the lymphocyte count decreased to 0.45 × 10^9^/L.

Because foscarnet was excessively myelosuppressive and could potentially impede hematopoietic recovery, he was transitioned to maribavir at a dose of 200 mg twice daily (20 mg/kg/day) on the 40th day after admission after obtaining consent from the family. ANC was 0.97 × 10^9^/L at the time of starting therapy. Four days later, CMV blood qPCR dropped below 500 copies/ml, and clinical status remained stable. At the same time, ANC increased to 1.56 × 10^9^/L, and the lymphocyte count increased to 1.29 × 10^9^/L.

Finally, the patient was discharged and oral maribavir was continued. The patient received maribavir for 2 weeks. For economic reasons, ganciclovir was administered after 2 weeks. CMV blood qPCR was <500 copies/mL on a regular test until 8 October 2024. No myelosuppression, elevated transaminase levels or renal impairment were found.

## Discussion

This case report describes the challenging course of CMV infection in a child with SIOD after renal transplantation. Following the ineffectiveness of GCV combined with foscarnet, which is associated with significant myelosuppression, maribavir demonstrated excellent anti-CMV efficacy and low myelosuppressive risk. The human immune response to CMV infection includes both innate and adaptive mechanisms (15). Among these, T lymphocytes play a major anti-CMV role (16). However, in our case, the child had SIOD, which is characterized by T-cell deficiency (12). Additionally, immunosuppressive drugs have been used to prevent rejection after renal transplantation, which broadly inhibit innate and adaptive defenses, including reduced effector cells, sustained reductions in immunoglobulins, and CMV-specific antibody levels (17, 18).

Under the “double strike” of T-lymphocyte deficiency and the administration of immunosuppressive drugs post-kidney transplantation to prevent rejection, this child exhibit severely compromised baseline immunity, making him highly susceptible to CMV infection or reactivation (19). At the same time, it poses a significant challenge for anti-CMV treatment.

Traditionally, GCV and foscarnet have been used as anti-CMV agents. However, one of the side effects of myelosuppression is further inhibition of immune function, especially foscarnet. Another side effect of foscarnet is renal damage because of its propensity to accumulate in renal tubules (20). Under the “triple strike” of primary disease and limited treatment, maribavir demonstrated significant efficacy with a lower risk of myelosuppression. Additionally, common adverse reactions to maribavir were not observed during treatment.

**Figure 2:**
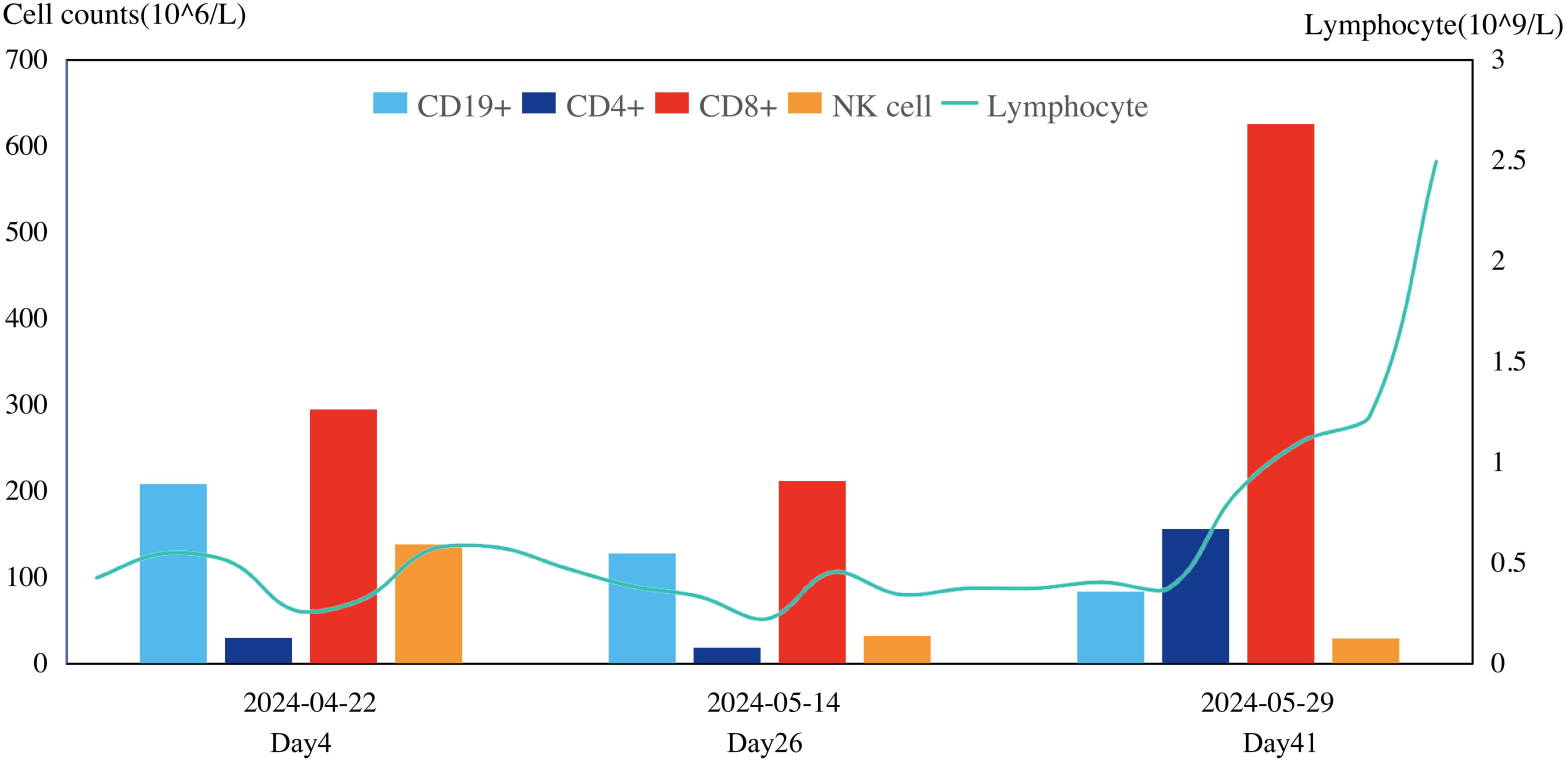
Change of immune cell.

In our case, CMV DNA was negative after discontinuation of foscarnet and administration of maribavir for four days. Maribavir exerts its anti-CMV effect by inhibiting the activity of UL97 protein kinase, which is an enzyme crucial for the replication of CMV and participates in several key processes in the CMV lifecycle (21, 22) (**Figure 2**). Although maribavir is administered twice daily and reaches a steady state within 2 days, whether the residual effects of foscarnet also play a role in antiviral activity is worth exploring. The long terminal phase elimination half-life of foscarnet (up to a mean of 88 h) is assumed to be the result of sequestration of the drug into the bone, but the shorter first- and second-phase half-lives (up to 1.4 h and 6.8 h) indicate that continuous or frequent short infusions are required to maintain adequate plasma drug concentrations. This suggests that foscarnet may have terminal half-life when maribavir antiviral therapy is initiated. Additionally, previous studies indicated that foscarnet concentrations of 0.1 umol/L to 0.5 umol/L inhibit CMV DNA polymerase activity by 50%. Unfortunately, we were unable to determine the drug concentration of foscarnet (23).

**Figure 3:**
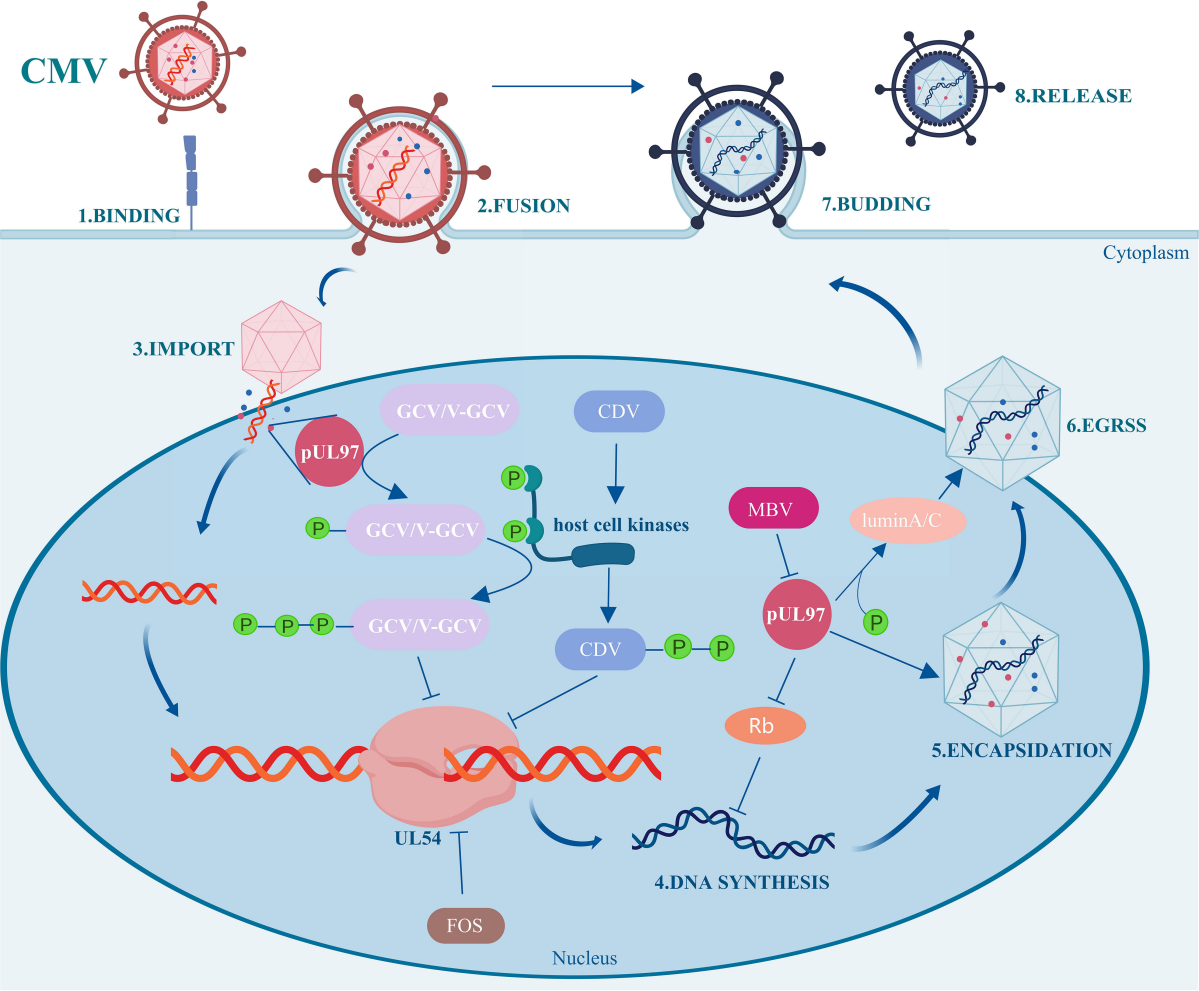
Mechanism diagram of maribavir against CMV.

Furthermore, after the cessation of anti-rejection drug therapy, the lymphocyte counts gradually increased, which suggested that the immune function had improved and provided strong support against CMV (**Figure 3**).

Currently, there is insufficient experience regarding the use of maribavir in pediatric patients. A literature review on the use of maribavir in pediatric patients has only three reported cases at the present time (24–26).

In reported cases, maribavir was used when conventional anti-CMV drugs, including foscarnet, GCV, valganciclovir, and cidofovir, were ineffective. Owing to limited research, there is no standardized dosage or duration of maribavir administration in children. The dosages used in the three reported cases ranged from 800 mg to 1,000 mg daily. Due to inconsistent underlying diseases, age, CMV-DNA copy number at baseline, and duration of treatment with maribavir, it seems difficult to summarize the appropriate dosage and duration of maribavir treatment in pediatric patients. However, it is not difficult to determine when the duration and dose increase, the mutation of CMV to resistant maribavir can be detected.

A 13-year-old girl with refractory pancytopenia developed CMV infection 6 weeks after bone marrow transplantation. She received maribavir at a dose of 400 mg twice daily for 7 weeks, and antiviral resistance testing revealed mutation L595S in the UL97 region, which has been reported to confer resistance to maribavir (26).

Similarly, a 4-year-old boy with Standard-risk B cell acute lymphoblastic lymphoma was infected with CMV on maintenance chemotherapy. Maribavir was administered at a dose of 200 mg every 8 hours, and the dose was increased gradually because CMV blood qPCR was persistently increased within 100 days. When the dose of maribavir was up to 1,000 mg daily, mutation T409M in CMV-UL97 was detected, which has also been described to confer resistance to maribavir after 10 days (25).

Therefore, it is important to determine the appropriate dosage and duration of maribavir treatment for children. In a clinical trial performed in adults, patients who responded to maribavir treatment similar among the groups (400 mg twice daily, 800 mg twice daily and 1,200 mg twice daily) (27). In another clinical trial performed in adults, the group that received maribavir at a dose of 400 mg twice daily showed a significant effect in eliminating CMV after 8 weeks when compared to the investigator-assigned treatment group (28). However, no high-quality clinical studies have examined the effects of maribavir dose and duration on efficacy in children. In our case, maribavir at a dose of 200 mg twice daily resulted in a reduction in the number of CMV-DNA copies from 693 to negative within four days, providing valuable insights for similar cases. Additional studies are required to explore the use of maribavir in children.

CMV resistance is another critical factor that affects efficacy (1). Like traditional anti-CMV drugs, including GCV, foscarnet, and cidofovir, maribavir also has resistance issues. Currently, well-defined CMV resistance mutations include the CMV-UL97 and CMV-UL54 genes, which encode phosphotransferases and DNA polymerases in the CMV-DNA replication process, respectively (29, 30). In pediatric cases, the mutation L595S–H411Y in the UL97 region was detected after seven weeks of maribavir treatment (25), and the mutation T409M in the UL97 region was detected after 232 days of maribavir treatment with a gradual increase in dose (26). Similarly, a clinical trial conducted in adult transplant recipients found that UL97 gene mutations were identified in 13 patients (52%), encompassing T409M in 10 patients and H411Y in three patients after maribavir treatment within 6 months (26). Resistance to maribavir in pediatric patients with CMV-infected has not yet been studied. Given the low barrier to resistance (25), monitoring of drug-resistant gene mutations may be necessary when maribavir is ineffective against CMV.

To our knowledge, this is the first case of maribavir being used for the treatment of refractory CMV viremia in a child with SIOD after renal transplantation. Under the combined strike of immunodeficiency, anti-rejection therapy after organ transplantation, and myelosuppression, it is important to find a balance between immunization and anti-CMV infection. We provided comprehensive clinical data, including diagnostic workup and complete treatment process, which will provide valuable insights for clinicians managing similar cases. Additionally, we also recognize that our study has some limitations. The relatively short follow-up period may not have fully captured the long-term effects of the treatment, and we were unable to obtain blood concentration data for foscarnet, which would have been valuable for assessing drug exposure and efficacy. Additionally, as a single-case study, our findings may not be generalizable to all pediatric patients. Future studies are needed to explore the long-term efficacy and safety of maribavir in pediatric patients.

## Conclusion

Maribavir-treated refractory-resistant CMV viremia and regulation of immune function in a child with SIOD after renal transplantation with favorable short-term efficacy. Further research is required to determine the optimal dosage, duration, long-term effects, and safety of maribavir in pediatric patients.

## Data Availability

The raw data supporting the conclusions of this article will be made available by the authors, without undue reservation.

## References

[1] Griffiths P Lumley S. Cytomegalovirus. Curr Opin Infect Dis Dec. (2014) 27:554–9. doi: 10.1097/qco.0000000000000107 25304390

[2] Humar A Lebranchu Y Vincenti F Blumberg EA Punch JD Limaye AP. The efficacy and safety of 200 days valganciclovir cytomegalovirus prophylaxis in high-risk kidney transplant recipients. Am J Transplant. May. (2010) 10:1228–37. doi: 10.1111/j.1600-6143.2010.03074.x 20353469

[3] Hodson EM Barclay PG Craig JC Jones C Kable K Strippoli GF. Antiviral medications for preventing cytomegalovirus disease in solid organ transplant recipients. Cochrane Database Syst Rev. (2005) 4):Cd003774. doi: 10.1002/14651858.CD003774.pub2 16235341

[4] Asakura M Ikegame K Yoshihara S Taniguchi S Mori T Etoh T. Use of foscarnet for cytomegalovirus infection after allogeneic hematopoietic stem cell transplantation from a related donor. Int J Hematol Sep. (2010) 92:351–9. doi: 10.1007/s12185-010-0657-y 20694532

[5] Ota R Hirata A. Relationship between renal dysfunction and electrolyte abnormalities in hematopoietic stem cell transplant patients treated with foscarnet. J Chemother Dec. (2021) 33:539–46. doi: 10.1080/1120009x.2021.1915074 34060436

[6] Razonable RR Humar A. Cytomegalovirus in solid organ transplant recipients-Guidelines of the American Society of Transplantation Infectious Diseases Community of Practice. Clin Transplant. Sep. (2019) 33:e13512. doi: 10.1111/ctr.13512 30817026

[7] Mehta Steinke SA Alfares M Valsamakis A Shoham S Arav-Boger R Lees L. Outcomes of transplant recipients treated with cidofovir for resistant or refractory cytomegalovirus infection. Transpl Infect Dis Jun. (2021) 23:e13521. doi: 10.1111/tid.13521 PMC9353742 33220125

[8] FDA approves first treatment for common type of post-transplant infection that is resistant to other drugs. (2021).

[9] Boerkoel CF Takashima H John J Yan J Stankiewicz P Rosenbarker L. Mutant chromatin remodeling protein SMARCAL1 causes Schimke immuno-osseous dysplasia. Nat Genet Feb. (2002) 30:215–20. doi: 10.1038/ng821 11799392

[10] Boerkoel CF O’Neill S André JL Benke PJ Bogdanovíć R Bulla M. Manifestations and treatment of Schimke immuno-osseous dysplasia: 14 new cases and a review of the literature. Eur J Pediatr. (2000) 159:1–7. doi: 10.1007/s004310050001 10653321

[11] Lipska-Ziętkiewicz BS Gellermann J Boyer O Gribouval O Ziętkiewicz S Kari JA. Low renal but high extrarenal phenotype variability in Schimke immuno-osseous dysplasia. PloS One. (2017) 12:e0180926. doi: 10.1371/journal.pone.0180926 28796785 PMC5552097

[12] Marin AV Jiménez-Reinoso A Mazariegos MS Román-Ortiz E Regueiro JR. T-cell receptor signaling in Schimke immuno-osseous dysplasia is SMARCAL1-independent. Front Immunol. (2022) 13:979722. doi: 10.3389/fimmu.2022.979722 36330520 PMC9623027

[13] Smith IL Cherrington JM Jiles RE Fuller MD Freeman WR Spector SA. High-level resistance of cytomegalovirus to ganciclovir is associated with alterations in both the UL97 and DNA polymerase genes. J Infect Dis Jul. (1997) 176:69–77. doi: 10.1086/514041 9207351

[14] Ljungman P Chemaly RF Khawaya F Alain S Avery R Badshah C. Consensus definitions of cytomegalovirus (CMV) infection and disease in transplant patients including resistant and refractory CMV for use in clinical trials: 2024 update from the transplant associated virus infections forum. Clin Infect Dis. (2024) 79:787–94. doi: 10.1093/cid/ciae321 PMC11426271 39041385

[15] Rowshani AT Bemelman FJ van Leeuwen EM van Lier RA ten Berge IJ. Clinical and immunologic aspects of cytomegalovirus infection in solid organ transplant recipients. Transplantation. (2005) 79:381–6. doi: 10.1097/01.tp.0000148239.00384.f0 15729162

[16] La Rosa C Diamond DJ. The immune response to human CMV. Future Virol. (2012) 7:279–93. doi: 10.2217/fvl.12.8 PMC3539762 23308079

[17] Carbone J. The immunology of posttransplant CMV infection: potential effect of CMV immunoglobulins on distinct components of the immune response to CMV. Transplantation. (2016) 100 Suppl 3:S11–8. doi: 10.1097/tp.0000000000001095 PMC4764014 26900990

[18] Kotton CN Torre-Cisneros J Aguado JM Alain S Baldanti F. Cytomegalovirus in the transplant setting: Where are we now and what happens next? A report from the International CMV Symposium 2021. Transpl Infect Dis. (2022) 24:e13977. doi: 10.1111/tid.13977 36271650 PMC10078482

[19] Aoyama Y Sugiyama S Yamamoto T. Anti-cytomegalovirus therapy: whether and when to initiate, those are the questions. Pharm (Basel). (2022). doi: 10.3390/ph15070797 PMC9325238 35890096

[20] Beaufils H Deray G Katlama C Dohin E Henin D Sazdovitch V. Foscarnet and crystals in glomerular capillary lumens. Lancet. (1990) 336:755. doi: 10.1016/0140-6736(90)92253-e 1975929

[21] Shannon-Lowe CD Emery VC. The effects of maribavir on the autophosphorylation of ganciclovir resistant mutants of the cytomegalovirus UL97 protein. Herpesviridae. (2010) 1:4. doi: 10.1186/2042-4280-1-4 21429239 PMC3050433

[22] Prichard MN. Function of human cytomegalovirus UL97 kinase in viral infection and its inhibition by maribavir. Rev Med Virol. (2009) 19:215–29. doi: 10.1002/rmv.615 PMC3777615 19434630

[23] Wagstaff AJ Bryson HM Foscarnet. A reappraisal of its antiviral activity, pharmacokinetic properties and therapeutic use in immunocompromised patients with viral infections. Drugs. (1994) 48:199–226. doi: 10.2165/00003495-199448020-00007 7527325

[24] Song E. Case Report: Approaches for managing resistant cytomegalovirus in pediatric allogeneic hematopoietic cell transplantation recipients. Front Pediatr. (2024) 12:1394006. doi: 10.3389/fped.2024.1394006 38884102 PMC11177687

[25] Fisher JE Mulieri K Finch E Ericson JE. Use of maribavir for multidrug resistant cytomegaloviremia in a pediatric oncology patient. J Pediatr Hematol Oncol. (2024) 46:e244–7. doi: 10.1097/mph.0000000000002841 PMC10956659 38447094

[26] Schubert A Ehlert K Schuler-Luettmann S Gentner E Mertens T Michel D. Fast selection of maribavir resistant cytomegalovirus in a bone marrow transplant recipient. BMC Infect Dis. (2013) 13:330. doi: 10.1186/1471-2334-13-330 23870704 PMC3720178

[27] Maertens J Cordonnier C Jaksch P Poiré X Uknis M Wu J. Maribavir for preemptive treatment of cytomegalovirus reactivation. N Engl J Med. (2019) 381:1136–47. doi: 10.1056/NEJMoa1714656 31532960

[28] Avery RK Alain S Alexander BD Blumberg EA Chemaly RF Cordonnier C. Maribavir for refractory cytomegalovirus infections with or without resistance post-transplant: results from a phase 3 randomized clinical trial. Clin Infect Dis. (2022) 75:690–701. doi: 10.1093/cid/ciab988 34864943 PMC9464078

[29] Chou S. Approach to drug-resistant cytomegalovirus in transplant recipients. Curr Opin Infect Dis. (2015) 28:293–9. doi: 10.1097/qco.0000000000000170 PMC4522269 26098499

[30] Halpern-Cohen V Blumberg EA. New perspectives on antimicrobial agents: maribavir. Antimicrob Agents Chemother. (2022) 66:e0240521. doi: 10.1128/aac.02405-21 35916518 PMC9487637

