# Supplementary material for: Case Report: Maribavir for refractory cytomegalovirus viremia after renal transplantation in a child with Schimke’s immune-osseous dysplasia
Source: Front Immunol. 2025 Apr 7;16:1521763. doi: 10.3389/fimmu.2025.1521763 (PMC12009931; doi:10.3389/fimmu.2025.1521763)
Supplement: Supplementary file 1 [file Table1.docx]

**Supplement table. The patient’s clinical time line**

| **Date** | | **Days Post-Admission (day)** | **Clinical Course** |
| --- | --- | --- | --- |
| 2024/4/19 | 1 | | Patient hospital admission due to recurrent abdominal pain and CMV virema (CMV qPCR was 7 x 10^4 copies/ml at other hospitals). Treated with ganciclovir after admission. |
| 2024/4/23 | 5 | | CMV blood qPCR increased to 1.01 x 10^5 copies/ml.  First antiviral resistance testing showed no resistance genes. |
| 2024/5/2 | 14 | | Began to successively discontinue the use of anti-rejection medications. |
| 2024/5/7 | 19 | | Chest CT showed interstitial pneumonia. CMV blood qPCR persistently increased to 1.35 x 10^5 copies/ml. |
| 2024/5/14 | 26 | | Alveolar lavage fluid CMV qPCR was 2.2 x 10^3 copies/ml, and CMV blood qPCR persistently increased to 1.64 x 10^5 copies/ml. Second antiviral resistance testing revealed emergence of HCMV variants with mutations (S676G), conferring GCV resistance.  Started combination antiviral therapy with Foscarnet. |
| 2024/5/28 | 40 | | CMV blood qPCR decreased to 693 copies/ml. Due to concerns of excessive myelosuppression from Foscarnet, transitioned to Maribavir at a dose of 200 mg twice daily (20 mg/kg/day). |
| 2024/6/1 | 44 | | CMV blood qPCR dropped below 500 copies/ml, accompanied by improvement in bone marrow suppression and stable clinical status, leading to the patient's discharge. |
| 2024/6/11 | +10 | | Completed 2 weeks of Maribavir treatment and continued Ganciclovir due to economic reasons. |
| 2024/12/28 | +211 | | Regular tests showed CMV blood qPCR less than 500 copies/ml. No myelosuppression, transaminases elevating, or renal impairment were found. |

CMV, cytomegalovirus; qPCR, quantitative PCR.; GCV, Ganciclovir.
